# Is cardiovascular fitness associated with structural brain integrity in midlife? Evidence from a population-representative birth cohort study

**DOI:** 10.18632/aging.104112

**Published:** 2020-10-21

**Authors:** Tracy d’Arbeloff, Megan Cooke, Annchen R. Knodt, Maria Sison, Tracy R. Melzer, David Ireland, Richie Poulton, Sandhya Ramrakha, Terrie E. Moffitt, Avshalom Caspi, Ahmad R. Hariri

**Affiliations:** 1 Department of Psychology and Neuroscience, Duke University, Durham, NC 27708, USA; 2 Center for Addiction Medicine, Department of Psychiatry, Massachusetts General Hospital, Boston, MA 02114, USA; 3 Harvard Medical School, Boston, MA 02115, USA; 4 New Zealand Brain Research Institute, Christchurch, New Zealand; 5 Department of Medicine, University of Otago, Christchurch, New Zealand; 6 Dunedin Multidisciplinary Health and Development Research Unit, Department of Psychology, University of Otago, Dunedin, NZ; 7 Social, Genetic, and Developmental Psychiatry Research Centre, Institute of Psychiatry, Psychology, and Neuroscience, King’s College London, De Crespigny Park, Denmark Hill, London, UK; 8 Department of Psychiatry and Behavioral Sciences, Duke University School of Medicine, Durham, NC 27708, USA; 9 Center for Genomic and Computational Biology, Duke University, Durham, NC 27708, USA

**Keywords:** aging, dementia, cardiovascular fitness, brain structure, cognitive decline

## Abstract

Improving cardiovascular fitness may buffer against age-related cognitive decline and mitigate dementia risk by staving off brain atrophy. However, it is unclear if such effects reflect factors operating in childhood (neuroselection) or adulthood (neuroprotection). Using data from 807 members of the Dunedin Study, a population-representative birth cohort, we investigated associations between cardiovascular fitness and structural brain integrity at age 45, and the extent to which associations reflected possible neuroselection or neuroprotection by controlling for childhood IQ. Higher fitness, as indexed by VO_2_Max, was not associated with average cortical thickness, total surface area, or subcortical gray matter volume including the hippocampus. However, higher fitness was associated with thicker cortex in prefrontal and temporal regions as well as greater cerebellar gray matter volume. Higher fitness was also associated with decreased hippocampal fissure volume. These associations were unaffected by the inclusion of childhood IQ in analyses. In contrast, a higher rate of decline in cardiovascular fitness from 26 to 45 years was not robustly associated with structural brain integrity. Our findings are consistent with a neuroprotective account of adult cardiovascular fitness but suggest that effects are not uniformly observed across the brain and reflect contemporaneous fitness more so than decline over time.

## INTRODUCTION

An aging global population has created an unprecedented need to preserve and prolong both physical and mental health [1–5]. Increases in average population age, as well as in longevity, can put extreme stress on health and social welfare systems; the growing size of adult populations is one of the largest factors associated with the major increase in age-related social and financial burden between 1990 and 2017 [2]. This is due, in part, to the accompanying increase in risk for aging-related dementias, a category that includes disorders such as Alzheimer’s disease, vascular dementia, and frontotemporal dementia. As the greatest risk factor for developing aging-related dementias is age, the most effective means to reduce burdens associated with these disorders is to extend years of life lived free of disease and associated disability [6–8]. However, no cure for dementia currently exists. Thus, there is a critical need for research into preventative measures or interventions to delay or prevent onset of symptoms. Of particular importance are large-scale efforts to identify effective interventions against cognitive decline, one of the most debilitating symptoms associated with aging-related dementias [9]. Declines in cognition precede any clinical diagnosis of dementia and are often a precursor to decreased quality of life [4, 10, 11].

One potential intervention against aging-related cognitive decline may be improving cardiovascular fitness [1, 4, 5]. As a measure of the physical work capacity of an individual, or the maximum rate at which the body can utilize oxygen [12–15], cardiovascular fitness reflects how efficiently the respiratory and circulatory systems are providing oxygenated blood to relevant musculature during active moments [12, 16]. Cardiovascular fitness can be measured or estimated in a variety of ways including self-report questionnaires, activity monitoring, or assessing maximum oxygen output, referred to as the volume of maximum oxygen uptake (VO_2_Max). VO_2_Max is considered the gold standard in the field for estimating cardiovascular fitness levels [17–19]. The advantages of good cardiovascular fitness for physical health are well documented, including increased mobility, increased quality of life, and decreased cardiovascular disease risk [14, 20–22]. However, emerging evidence suggests cardiovascular fitness may also have beneficial effects on brain structure and cognitive function [4, 23–25].

Higher cardiovascular fitness has been associated with better executive function and decreased rates of cognitive decline [10, 25–27]. Higher cardiovascular fitness has also been linked with structural brain integrity including increased cortical thickness and surface area, as well as increased gray matter volume of subcortical structures including the hippocampal formation, which supports memory and cognitive processes disrupted in aging-related dementias [5, 25, 28, 29]. These associations suggest that cardiovascular fitness may help buffer against aging-related neurocognitive decline, a phenomenon often referred to as neuroprotection. Such possible neuroprotective effects are important because aging-related declines in cognitive ability are tied with structural atrophy of the brain, which manifests years in advance of clinical symptoms and diagnosis [1, 8, 27]. Individual differences in structural brain integrity may thus provide a way to gauge risk for aging-related dementias.

Prior research has already documented that the brains of individuals who later develop dementia exhibit more age-related atrophy of gray matter volume in subcortical regions before the onset of cognitive impairments [30]. Similarly, decreased cortical thickness has been observed in the brains of those who later go on to develop aging-related dementias [30–32]. In addition, decreased cortical thickness has been correlated with the severity of clinical impairment and cognitive decline during the early stages of dementia [30, 33]. Thus, reduced structural brain integrity may represent part of the process toward accelerated cognitive decline and risk for aging-related dementias. Consequently, improving cardiovascular fitness as a means of mitigating negative age-related outcomes through slowing or reversing age-related brain atrophy has been heralded as a promising avenue of research.

However, recent studies suggest that at least some of the cognitive benefits associated with adult cardiovascular fitness identified in cross-sectional studies may be better explained by factors present since childhood [4, 15, 21, 23]. In other words, while better cardiovascular fitness may be correlated with better adult cognition, this correlation cannot be assumed to indicate causation. Instead, early life factors—often referred to as neuroselective influences—may underlie the associations observed later in life. One measure used to test for neuroselection is intelligence quotient (IQ) testing, which is a reliable and valid indicator of the brain’s cognitive function and is stable across the lifespan. According to the “neuroselection” model, people who began life with better cognitive function may have subsequently made certain choices (e.g., healthy diet and nutrition, regular exercise, more sports participation) or had certain advantages (e.g., safer neighborhoods for outside activities, access to healthier foods, better healthcare) that have carried forward to adulthood. These lifestyle differences, in turn, may lead to higher adult cardiovascular fitness. In this neuroselection scenario, higher cognitive function precedes higher cardiovascular fitness.

Associations between adult cardiovascular fitness and structural brain integrity may also be downstream effects of neuroselection as both adult fitness and adult brain structure are independently associated with neuroselective variables such as childhood IQ [4, 23, 34, 35]. However, few observational studies of structural brain integrity and adult cardiovascular fitness have taken potential neuroselective factors into account [4, 23, 25]. This is a critical step before proposing fitness interventions as one way to preserve brain structure as a prelude to slowing age-related cognitive decline and mitigating risk for associated aging-related dementias. Cross-sectional association research that neglects to control for neuroselective variables may yield overestimates of the potential effect size that fitness interventions can have on not only structural brain integrity but also other aging phenotypes of interest (e.g., risk for aging-related dementias).

It should be noted that adult cardiovascular fitness is not a static measure. Cardiovascular fitness, like brain integrity and cognitive ability, declines with age. For example, by age 65 cardiovascular fitness capacity may be up to 40% lower than it was in young adulthood [22]. This aging-related decrease in cardiovascular fitness can contribute to increased risk of disability, loss of independence, and reduced quality of life [14, 23, 36]. Longitudinal estimates of the rate of decline in cardiovascular fitness during adulthood may better indicate trends in physiological health associated with increased risk for aging-related disability than cross-sectional estimates. However, few studies have access to such longitudinal repeated measures data needed to directly test whether the rate of decline in individuals’ cardiovascular fitness is associated with structural brain integrity outcomes. It is possible that individual variation in the rate of decline in cardiovascular fitness over time, after accounting for neuroselective confounds, might be independently associated with individual variation in structural brain integrity. Testing for such an association may offer additional insight into the complex relationship between cardiovascular fitness, structural brain integrity, and aging-related cognitive decline and dementia risk.

Here, we analyzed data from 807 members of the population representative Dunedin Study birth cohort to test hypotheses about associations between structural brain integrity at age 45 and cardiovascular fitness at age 45 as well as the rate of decline in cardiovascular fitness across two decades of adulthood. Specifically, we tested the hypotheses that higher cardiovascular fitness was positively associated with greater cortical thickness and surface area as well as subcortical gray matter volume—with a specific focus on the hippocampal formation, given its central role in memory and executive control processes disrupted in aging-related dementias. Although these measures of structural brain integrity show common patterns of age-related atrophy, distinguishing between each of these three measures in analyses affords mapping of possibly different trajectories that could reflect independent aging-related pathophysiology [37]. Critically, we evaluated the potential confounding effects of tested childhood IQ on any observed associations in midlife to disentangle potential neuroselective from neuroprotective effects.

## RESULTS

Supplementary Table 2 provides a summary of the means and distributions of the primary variables for the 807 Study members included in our analyses. Study members had an average VO_2_Max score of 26.94 ± 7.42 mL/min/kg at age 45, and VO_2_Max declined, on average, by 3.16 mL/min/kg between the ages 26 to 45 years (Supplementary Figure 2). At age 45, Study members had an average cortical thickness of 2.56 ± 0.09 mm and an average total surface area of 1850 ± 161.7 cm^2^. As expected, there were significant sex differences in mean VO_2_Max at age 45, in the rate of VO_2_Max decline, and in global brain structure (Supplementary Table 2) [38].

### Is cardiovascular fitness at age 45 associated with structural brain integrity?

**Figure 1:**
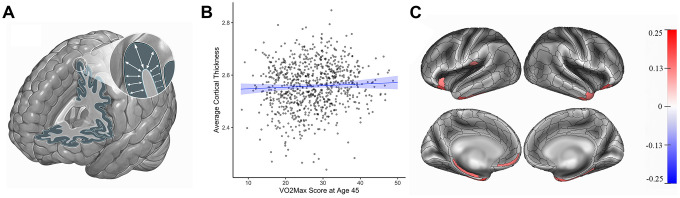
**Cortical thickness (mm) and cardiovascular fitness (mL/min/kg) at age 45.** (**A**) Cortical thickness. (**B**) Graph showing the correlation between average cortical thickness (mm, y-axis) and VO_2_Max (mL/min/kg; x-axis). Average cortical thickness was unrelated to the VO_2_Max scores of Study members (β=0.05, 95% CI = -0.04 to 0.14 *p*=0.28). (**C**) Study members with higher VO_2_Max scores, however, had increased parcel-wise thickness in multiple regions encompassing anterior temporal cortex, parahippocampal gyrus, and prefrontal cortex. Color bar on the right of the figure indicates a possible range of βs from -.25 to +.25. The color of each parcel on the simulated brains represents the associated effect size with cardiovascular fitness. Parcels colored in gray did not remain significantly associated with cardiovascular fitness after adjusting for multiple comparisons. All results pictured are adjusted for sex. mL/min/kg = milliliters per minute per kilogram; VO_2_Max = volume of maximum oxygen uptake; β = standardized coefficient; CI = confidence interval.

Study members with higher cardiovascular fitness at age 45 did not have significantly different average cortical thickness than study members with lower cardiovascular fitness (β=0.05, 95% CI = -0.04 to 0.14, *p*=0.28). However, parcel-wise analyses showed that Study members with higher cardiovascular fitness did have significantly thicker cortex in 16 subregions largely falling within bilateral anterior temporal cortex, parahippocampal gyrus, and prefrontal cortex (βs range from 0.14 to 0.25, *p* values range from = 0.04 to <0.001 after correcting for multiple testing, Figure 1). For βs of all 360 parcels see Supplementary Figure 3. Study members with higher cardiovascular fitness at age 45 did not have significantly different total surface area than study members with lower cardiovascular fitness (β=-0.03, 95% CI = -0.10 to 0.045, *p*=0.45). Likewise, parcel-wise analyses of surface area did not reveal any significant regional associations with cardiovascular fitness at age 45. For βs of all 180 bilateral parcels see Supplementary Figure 4.

**Figure 2:**
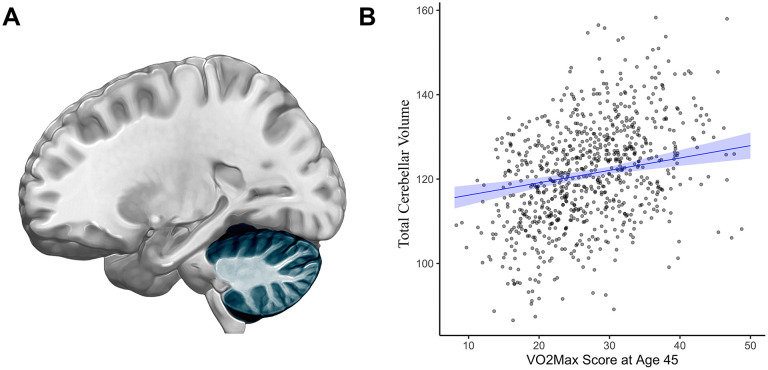
**Cerebellar cortex gray matter volume (cm^3^) and cardiovascular fitness (mL/min/Kg) at age 45.** (**A**) In addition to its well-known role in motor coordination and balance, the cerebellar cortex (highlighted in blue) and particularly the cerebellar cortex contribute to higher-order functions including learning and memory as well as executive control [44, 45] (**B**) Graph showing the correlation between average cerebellar cortex volume (cm^3^, y-axis) and VO_2_Max (mL/min/kg; x-axis). Study members with higher cardiovascular fitness at age 45 had greater gray matter volume of the cerebellar cortex (β= 0.17, 95% CI = 0.1 to 0.25, *p*<0.001). Results pictured are adjusted for sex. cm = centimeters; mL/min/kg = milliliters per minute per kilogram; VO_2_Max = volume of maximum oxygen uptake; β = standardized coefficient; CI = confidence interval.

**Figure 3:**
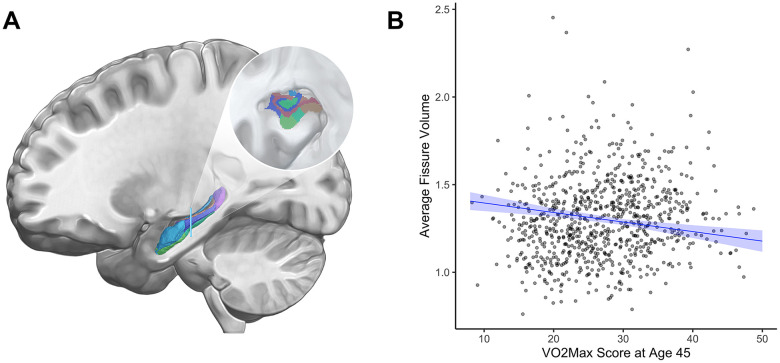
**Hippocampal fissure volume (cm^3^) and cardiovascular fitness (mL/min/Kg) at age 45.** (**A**) The hippocampal formation, which supports episodic memory and cognition, is comprised of multiple subregions (inset) that follow independent aging trajectories [47]. The hippocampal fissure is an interstitial space separating two of these subregions, the dentate gyrus and the subiculum. (**B**) Graph showing the correlation between average hippocampal fissure volume (cm^3^, y-axis) and VO_2_Max (mL/min/kg; x-axis). Study members with higher cardiovascular fitness at age 45 had smaller mean volume of the hippocampal fissure (β = -0.18, 95% CI = -0.26 to -0.09, *p*<0.001). Results pictured are adjusted for sex. cm^3^ = centimeters cubed; mL/min/kg = milliliters per minute per kilogram; VO_2_Max = volume of maximum oxygen uptake; β = standardized coefficient; CI = confidence interval.

Analyses of subcortical gray matter volume revealed only one significant association: Study members with higher cardiovascular fitness at age 45 had greater gray matter volume of cerebellar cortex (β= 0.17, 95% CI = 0.1 to 0.25, *p*<0.001, Figure 2). While cardiovascular fitness was not significantly associated with mean hippocampal gray matter volume (β=-0.05, 95% CI = -0.14 to 0.026, *p*=0.18), analyses of hippocampal formation subregions revealed a significant association with lesser volume of the hippocampal fissure after correcting for multiple comparisons (β = -0.18, 95% CI = -0.26 to -0.09, *p*<0.001, Figure 3). For βs of all bilateral subcortical regions see Supplementary Figures 5, 6.

### Are changes in cardiovascular fitness over time associated with structural brain integrity?

**Figure 4:**
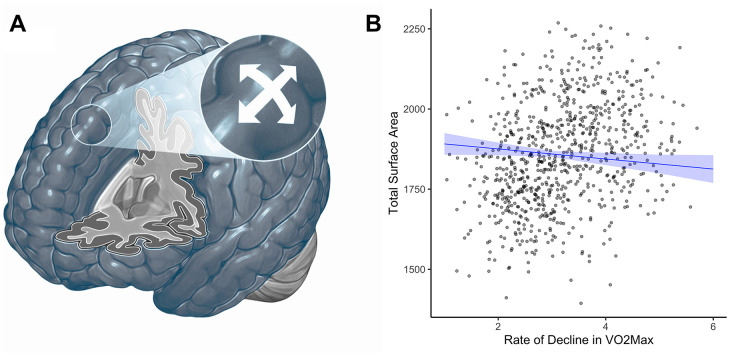
**Cortical surface area (cm^2^) at age 45 and rate of decline in VO_2_Max scores (mL/min/Kg) from age 26 to 45.** (**A**) Surface area. (**B**) Graph showing the correlation between total surface area (cm^2^, y-axis) and the rate of decline in VO_2_Max (average decrease in mL/min/kg between each wave of data collection; x-axis). Study members with VO_2_Max scores declining at a faster rate over time (i.e., larger slopes) did have slightly smaller total surface area (β = -0.09, 95% CI = -0.17 to -0.004, *p*=0.04). There were no regionally-specific associations. cm^3^ = centimeters cubed; VO_2_Max = volume of maximum oxygen uptake; mL/min/kg = milliliters per minute per kilogram; β = standardized coefficient; CI = confidence interval.

Study members whose cardiovascular fitness was declining at a slower rate across time did not have different average cortical thickness than study members whose cardiovascular fitness was declining at a comparatively faster rate across time (β = -0.002, 95% CI = -0.09 to 0.09, *p*=0.97). Likewise, parcel-wise analyses did not reveal any significant regional associations in cortical thickness with rates of decline in cardiovascular fitness over time. For βs of all 360 parcels see Supplementary Figure 7. Study members whose cardiovascular fitness was declining at a slower rate over time did have slightly greater total surface area at age 45 (β = -0.09, 95% CI = -0.17 to -0.004, *p*=0.04; Figure 4). Parcel-wise analyses did not show any regionally-specific associations. For βs of all bilateral 360 parcels see Supplementary Figure 8.

Analyses of regional subcortical volume, however, revealed no significant associations. Likewise, Study members whose cardiovascular fitness was declining at slower rates did not demonstrate any significant differences in mean hippocampal volume than study members whose cardiovascular fitness was declining at a comparatively faster rate across time (β = -0.05, 95% CI = -0.14 to 0.03, *p*=0.19) or subregional volumes of the hippocampal formation. For βs of all subcortical regions see Supplementary Figures 9, 10.

### Does childhood IQ account for associations between cardiovascular fitness and structural brain integrity?

The addition of childhood IQ to the above analyses did not change any results. Specifically, the associations between cardiovascular fitness at age 45 and parcel-wise cortical thickness remained significant when covarying for childhood IQ (Supplementary Figure 11). Similarly, cardiovascular fitness at age 45 remained significantly associated with mean cerebellar cortex gray matter volume and hippocampal fissure volume when covarying for childhood IQ (Supplementary Figure 12) as did the association between rate of change in cardiovascular fitness and total cortical surface area.

## DISCUSSION

In a population-representative birth cohort aged 45 years, we investigated associations between cardiovascular fitness and structural brain integrity. Broadly, we observed no uniform associations when comparing across all measures of brain structure. For example, there were no associations between midlife cardiovascular fitness, and measures of total surface area, average cortical thickness, or subcortical gray matter volume. In contrast, age-45 cardiovascular fitness was associated with thicker cortex in bilateral temporal and prefrontal subregions as well as greater gray matter volume of cerebellar cortex and lesser volume of the hippocampal fissure. Regardless of their specific nature, none of the observed associations were affected by the inclusion of childhood IQ in analyses, which is consistent with a neuroprotective model of cardiovascular fitness. We next compare our findings with prior studies before considering the specific patterns observed in our analyses in detail.

### Comparison with prior studies

Prior studies have reported a number of cross-sectional associations between cardiovascular fitness and structural brain integrity, although it is important to note that these findings have been mixed and often lack spatial consistency in the brain [24, 39]. Higher cardiovascular fitness has been associated with greater total and regional cortical thickness and surface area as well as increased gray matter volume of subcortical structures including the hippocampus [5, 25, 28, 29]. Additionally, higher cardiovascular fitness has been associated with greater gray matter volume of prefrontal and temporal cortex as well as cerebellar cortex [40]. Our analyses provide partial support for these prior links. While we did find an association between cardiovascular fitness at age 45 and cortical thickness, this was limited to prefrontal and temporal subregions. We did not find any cross-sectional associations with average cortical thickness or with any measures of surface area. Our cross-sectional analyses did not implicate the hippocampus directly as in prior studies. They did, however, yield associations between cardiovascular fitness and subregional volumes of the broader hippocampal formation including the parahippocampal gyrus and hippocampal fissure, which we discuss below.

These differences may reflect a number of factors. For example, our cross-sectional data are from Study members in midlife, whereas prior studies of fitness have often included data from older adults or those already showing clinical signs of decline. Heterogeneity of fitness measures in the published literature may also have contributed to inconsistent results. Our analyses utilized VO_2_Max, a valid physiological measure of cardiovascular fitness. Many prior studies, in contrast, have relied on self-report data of activity levels or hours of exercise per week. Cardiovascular fitness and exercise are not necessarily correlated [15], resulting in divergent associations with brain structure. Lastly, our high-resolution MRI data afforded analyses of brain structures (e.g., hippocampal formation subregions) that may not have been possible to identify with lower-resolution data collected in prior studies. The inability to resolve such subregional anatomy may contribute to the generally inconsistent associations reported between fitness and hippocampal gray matter volume [24].

### Cardiovascular fitness at age 45

The findings of higher cardiovascular fitness at age 45 and thicker cortex in bilateral temporal and prefrontal subregions represents one set of replicated associations [5, 40]. The prefrontal cortex associations included the medial and orbitofrontal cortex, which are important for higher-order executive functions [41]. The temporal cortex associations included the parahippocampal gyrus and temporal pole, which are important for episodic memory and semantic knowledge [4, 8, 27]. Aging-related cortical thinning in frontotemporal regions has been associated with cognitive decline in both healthy individuals and those with Alzheimer’s disease [31–33]. Moreover, frontotemporal atrophy is a common archetype of pathological aging and is considered one of the main causes of dementia [42, 43]. In line with this, recent research suggests that individuals with greater frontotemporal atrophy are at an increased risk for future clinical diagnoses of Alzheimer’s disease [42]. Our observation of associations between cross-sectional cardiovascular fitness and cortical thickness in prefrontal and temporal subregions suggests that possible buffering effects of cardiovascular fitness on cognitive decline may operate through these structural brain features.

Higher cardiovascular fitness at age 45 was also associated with greater gray matter volume of the cerebellar cortex. In addition to its well-known role in motor coordination and balance, the cerebellar cortex broadly and the cerebellar cortex specifically contribute to higher-order functions including learning and memory as well as executive control [44, 45]. These latter functions of the cerebellar cortex are linked with bidirectional communication with subregions of the prefrontal cortex [45]. As both gross-motor and higher-level executive functioning are implicated in neurodegenerative diseases, the cerebellar cortex has emerged as a brain area of interest in aging research [40, 44]. The cerebellar cortex in particular remains relatively spared in the early-stages of aging-related dementias, including Alzheimer’s disease, and some research suggests it may act as an early compensatory mechanism helping to maintain brain resilience when other brain regions begin to show aging-related pathology [40]. Our current findings extend this view of the cerebellar cortex by suggesting it may be a further target of neuroprotection associated with cardiovascular fitness.

Study members with higher cardiovascular fitness at age 45 had lesser volume of the hippocampal fissure, an interstitial space separating two hippocampal formation subregions, the dentate gyrus and subiculum [46]. Recent research suggests that increases in hippocampal fissure volume are an early indicator of greater gray matter atrophy of the hippocampal formation [46, 47]. This is consistent with the above association between cardiovascular fitness and increased cortical thickness of the parahippocampal gyrus, which is immediately adjacent to the hippocampal fissure (i.e., increased cortical thickness is further manifest as decreased interstitial space encompassing these same regions). Given the absence of a significant association between cardiovascular fitness and subcortical gray matter volume, including that of the hippocampus proper, our findings suggest that, at least in midlife, cardiovascular fitness may be more strongly associated with the integrity of cortical in comparison with subcortical structures. Thus, while observable associations between cardiovascular fitness and total gray matter volume of the hippocampus as well as other subcortical structures may emerge in later life, our findings suggest that studies in midlife would benefit from analyses of subregional volumes that capture cortical atrophy (e.g., hippocampal fissure).

### Change in cardiovascular fitness in adulthood

Access to nearly two decades of cardiovascular fitness measurements afforded the novel opportunity to map individual rates of decline in cardiovascular fitness between ages 26 and 45 years onto structural brain integrity. While Study members whose fitness was declining at a slower rate did show slightly greater total cortical surface area, there were no associations with total or regional cortical thickness or any subcortical regional volumes. However, as recent research has demonstrated that age-related changes in surface area tend to be seen later in life, the lack of associations between the rate of change of fitness over time and brain structure seen at age 45 may become more relevant as Study members age [37].

### Non-uniformity of associations

While not hypothesized, divergent associations between different measures of structural brain integrity and cardiovascular fitness may not be altogether unexpected. Measures of cortical thickness and surface area are genetically, phylogenetically, and ontogenetically distinct from one another and follow discrete aging trajectories that are each shaped by different biological mechanisms [37, 43, 48]. For example, some age-related decreases in cortical thickness are likely associated with changes in the microstructural integrity of the cortical mantel while decreases in surface area are likely more attributable to intracortical or subcortical volumetric changes [37]. Thus, one possibility for the observed non-uniformity is that the rate at which an individual’s cardiovascular fitness declines may be associated with biological and physiological aging processes that are not captured in cross-sectional measures of cardiovascular fitness or that may be an indication of widespread decline in different aspects of physical health [35, 37]. Future studies are needed to differentiate the varying physiological processes underlying both aspects of cardiovascular fitness and subsequent associations with structural brain integrity. For example, other relevant age-sensitive physiological measures such as ApoB100/A1 ratio or body mass index (both of which are correlated with VO_2_max scores [7]) could be used to differentiate processes associated with cross-sectional fitness from those associated with rates of decline in fitness. These orthogonal processes may then help shed light on the different physiological mechanisms underlying associations between facets of fitness and brain structure. Understanding the processes contributing to associations between different structural measures and different aspects of fitness could help tailor future interventions to increase efficacy in targeting changes in the brain.

### Neuroprotection versus neuroselection

We were able to test whether associations between adult cardiovascular fitness and structural brain integrity were confounded by childhood IQ—a marker of cognitive ability that remains stable across the life course. Attenuation of observed associations by the inclusion of childhood IQ would have suggested that links between adult cardiovascular fitness and structural brain integrity were better explained by neuroselection. However, the addition of childhood IQ to our analyses did not attenuate or significantly affect any observed associations. Thus, the relationships we observed between cardiovascular fitness and structural brain integrity do not appear to be due to higher cognitive ability as a child.

Ruling out childhood IQ is an important step towards validating the potential utility of cardiovascular fitness as a neuroprotective mechanism that may buffer against aging-related brain atrophy. It is important to note, however, that childhood IQ is only one of many possible neuroselective variables. There are other possible neuroselective confounds such as childhood brain integrity. Unfortunately, due to the relative recency of MRI technology, no longitudinal cohort in midlife or later has childhood brain scans. However, as more recent longitudinal studies (e.g., NIH ABCD Study) progress, these measures may become available for analyses. Meanwhile, future observational studies could systematically rule out possible neuroselection (e.g., by controlling for childhood IQ as we have) to further bolster a neuroprotective role of cardiovascular fitness.

## CONCLUSIONS

Our findings suggest that associations between adult cardiovascular fitness and structural brain integrity are consistent with neuroprotective effects. However, such associations may be non-uniform and reflect specific features of both fitness (i.e., contemporaneous vs. rate of decline) and brain structure (i.e., global vs. regional; thickness vs. surface area). Notwithstanding the inherent limitations of cross-sectional associations, our findings suggest that future intervention studies investigating links between cardiovascular fitness and age-related brain atrophy should examine multiple indices of structural brain integrity to better evaluate possible neuroprotective effects.

## MATERIALS AND METHODS

### Study design and population

Data were derived from the Dunedin Study, a longitudinal investigation of health and behavior in a population representative birth cohort. Study members (N=1,037; 91% of eligible births; 52% male) are all individuals born between April 1972 and March 1973 in Dunedin, New Zealand (NZ), who were eligible based on residence in the province and who participated in the first assessment at age 3 years [49]. The cohort represented the full range of socioeconomic status (SES) in the general population of NZ’s South Island and as adults matched the NZ National Health and Nutrition Survey on key adult health indicators (e.g., body mass index (BMI), smoking, GP visits) and the NZ Census of citizens of the same age on educational attainment [50]. The cohort is primarily white (93%), matching South Island demographics [49]. Data were available at birth and assessments were carried out at ages 3, 5, 7, 9, 11, 13, 15, 18, 21, 26, 32, 38, and most recently (completed April 2019) 45 years, when 94.1% (N=938) of the 997 participants still alive took part. Of the 938 Study members participating in the latest assessment, 875 (93%) completed MRI scanning. Attrition analyses (see Supplementary Figure 1 for details) revealed that scanned Study members resembled still-living cohort members on childhood IQ and their family-of-origin’s socio-economic status. The relevant ethics committees approved each phase of the Study and written informed consent was obtained from all Study members before their participation.

### Predicted maximum oxygen uptake

Cardiovascular fitness was predicted by measuring heart rate in response to a submaximal exercise test on a friction-braked cycle ergometer at ages 26, 32, 38, and 45 years. Depending on the extent to which heart rate increased during a 2-minute 50W warm-up, the workload was adjusted to elicit a steady heart rate in the range of 130–170 beats per minute. After a further 6-minute constant power output stage, the maximum heart rate was recorded and used to predict maximum oxygen uptake (VO_2_Max) adjusted for body weight in milliliters per minute per kilogram (mL/min/kg) according to standard protocols [51].

### Growth curve model of fitness

Latent growth curve analysis (LGCA) is a type of structural equation modelling used to understand individual differences in within-subject change over time. In LGCA the observed repeated measures serve as multiple indicators on latent factors. These latent factors represent the intercept and slope of unobserved growth curves. We used the four time points of VO_2_Max (i.e., ages 26, 32, 38, 45) as indicators in three latent growth curve models: 1) intercept only, 2) intercept and linear slope, and 3) intercept, linear slope, and quadratic slope. All models were fit in R using the lavaan package [52]. Model fit was assessed using the chi-square test, root mean square error of approximation (RMSEA), the Tucker-Lewis Index (TLI), and the comparative fit index (CFI) [53, 54]. The intercept and linear slope model generated the best fit to our data based on the following fit statistics: TLI = 0.987, CFI = 0.989, and RMSEA = 0.093 as well as a visual examination of the data (Supplementary Figure 2). For a comparison of the fit statistics of all three models tested, see Supplementary Table 1.

### Measurement of cognitive ability

Cognitive ability was assessed at ages 7, 9, and 11 years using the Wechsler Intelligence Scale for Children Revised (WISC-R; Wechsler, 1974)). The mean WISC-R was computed for these three childhood assessments and used as the measure of childhood IQ in all analyses [55].

### Image acquisition

Study members were scanned at the Pacific Radiology Group imaging center in Dunedin, NZ using a MAGNETOM Skyra 3T scanner (Siemens Healthcare GmbH) equipped with a 64-channel head/neck coil. High resolution T1-weighted images were obtained using an MP-RAGE sequence with the following parameters: TR = 2400 ms; TE = 1.98 ms; 208 sagittal slices; flip angle, 9°; FOV, 224 mm; matrix =256×256; slice thickness = 0.9 mm with no gap (voxel size 0.9×0.875×0.875 mm); and total scan time = 6 min and 52 s. 3D T2-weighted fluid-attenuated inversion recovery (FLAIR) images were obtained with the following parameters: TR = 8000 ms; TE = 399 ms; 160 sagittal slices; FOV = 240 mm; matrix = 232×256; slice thickness = 1.2 mm (voxel size 1.2×0.9×0.9 mm); and total scan time = 5 min and 38 s. Additionally, a gradient echo field map was acquired with the following parameters: TR = 712 ms; TE = 4.92 and 7.38 ms; 72 axial slices; FOV = 200 mm; matrix = 100×100; slice thickness = 2.0 mm (voxel size 2 mm isotropic); and total scan time = 2 min and 25 s.

### Image processing

Structural MRI data were analyzed using the Human Connectome Project (HCP) minimal preprocessing pipeline as extensively detailed elsewhere [56]. Briefly, T1-weighted and FLAIR images were processed through the PreFreeSurfer, FreeSurfer, and PostFreeSurfer pipelines. T1-weighted and FLAIR images were corrected for readout distortion using the gradient echo field map, coregistered, brain-extracted, and aligned together in native T1 space using boundary-based registration [57]. Images were then processed with a custom FreeSurfer recon-all pipeline optimized for structural MRI with higher resolution than 1 mm isotropic. Finally, recon-all outputs were converted into CIFTI format and registered to common 32k_FS_LR mesh using MSM-sulc [58]. For each subject the mean cortical thickness and surface area were then extracted from each of the 360 cortical areas in the HCP-MPP1.0 parcellation [59]. Additionally, gray matter volumes for eight subcortical structures and cerebellar cortex were extracted from the FreeSurfer "aseg" parcellation (v. 6.0). Given prior focus on associations between cardiovascular fitness and the structural integrity of the hippocampal formation [8, 28, 47], we further examined associations between cardiovascular fitness and gray matter volumes of 22 subregions of the hippocampal formation [60].

### Statistical analyses

Outputs of the minimal preprocessing pipeline were visually inspected for accurate surface generation by examining each subject’s myelin map, pial surface, and white matter boundaries. Of the 875 study members for whom data were available, 5 were excluded due to major incidental findings or previous injuries (e.g., large tumors or extensive damage to the brain/skull), 8 due to missing FLAIR or field map scans, and 1 due to poor surface mapping. Of the 861 study members with available imaging data, 54 were missing VO_2_Max data at age 45, leaving a final sample size of 807 for analyses.

All analyses were conducted in R version 3.6.0 [61]. To investigate the cross-sectional relationship between cardiovascular fitness and structural brain integrity in midlife, we first tested the association between Study members’ VO_2_Max at age 45 and global structural measures including total surface area and average cortical thickness using linear regression. Regional associations with cortical thickness and surface area were then examined by regressing values for all 360 regions comprising the parcellation scheme described above [59] on age-45 VO_2_max. We then investigated whether individuals’ changes in fitness over time were associated with their midlife brain structure by repeating all regressions using individual linear growth slopes extracted from the growth curve modelling of VO_2_max (described above) as the variable of interest in the regressions. Intercept was included as a covariate in the model to account for baseline effects. These analyses were next repeated for the mean gray matter volume of cerebellar cortex and 8 subcortical structures including the hippocampus as well as 22 hippocampal subregional volumes. For each set of analyses (i.e., cortical thickness, surface area, subcortical volume), we corrected for multiple comparisons using a false discovery rate (FDR) procedure [62]. Finally, to test whether variation in pre-existing cognitive function (i.e., neuroselection) could explain associations found between cardiovascular fitness, change in cardiovascular fitness, and structural brain integrity, we repeated all analyses with childhood IQ included as a covariate.

We did not covary for global structural measures during regional analyses (i.e., total surface area, average cortical thickness) because we were interested in specific regional associations with cardiovascular fitness as well as regional contributions to global effects. Moreover, covarying for global measures removes global effects and has a consequence of artificially creating negative correlations between regional associations [63]. However, sex was included as a covariate in all analyses. The premise and analysis plan for this project were registered at https://sites.google.com/site/dunedineriskconceptpapers/home/dunedin-approved. Analyses reported here were checked for reproducibility by an independent data-analyst, who recreated the code by working from the manuscript and applied it to a fresh dataset.

### Additional information

The premise and analysis plan for this project were registered at https://sites.google.com/site/dunedineriskconceptpapers/home/dunedin-approved.

The dataset reported in the current article is not publicly available due to lack of informed consent and ethical approval but is available on request by qualified scientists. Requests require a concept paper describing the purpose of data access, ethical approval at the applicant's institution, and provision for secure data access. We offer secure access on the Duke University and Otago University campuses. All data analysis scripts and results files are available for review.

## Supplementary Material

Supplementary Figures

Supplementary Tables
