# Supplementary material for: Is cardiovascular fitness associated with structural brain integrity in midlife? Evidence from a population-representative birth cohort study
Source: Aging (Albany NY). 2020 Oct 21;12(20):20888–914. doi: 10.18632/aging.104112 (PMC7655208; doi:10.18632/aging.104112)
Supplement: Supplementary Figures [file aging-12-104112-s001..pdf]

SUPPLEMENTARY FIGURES

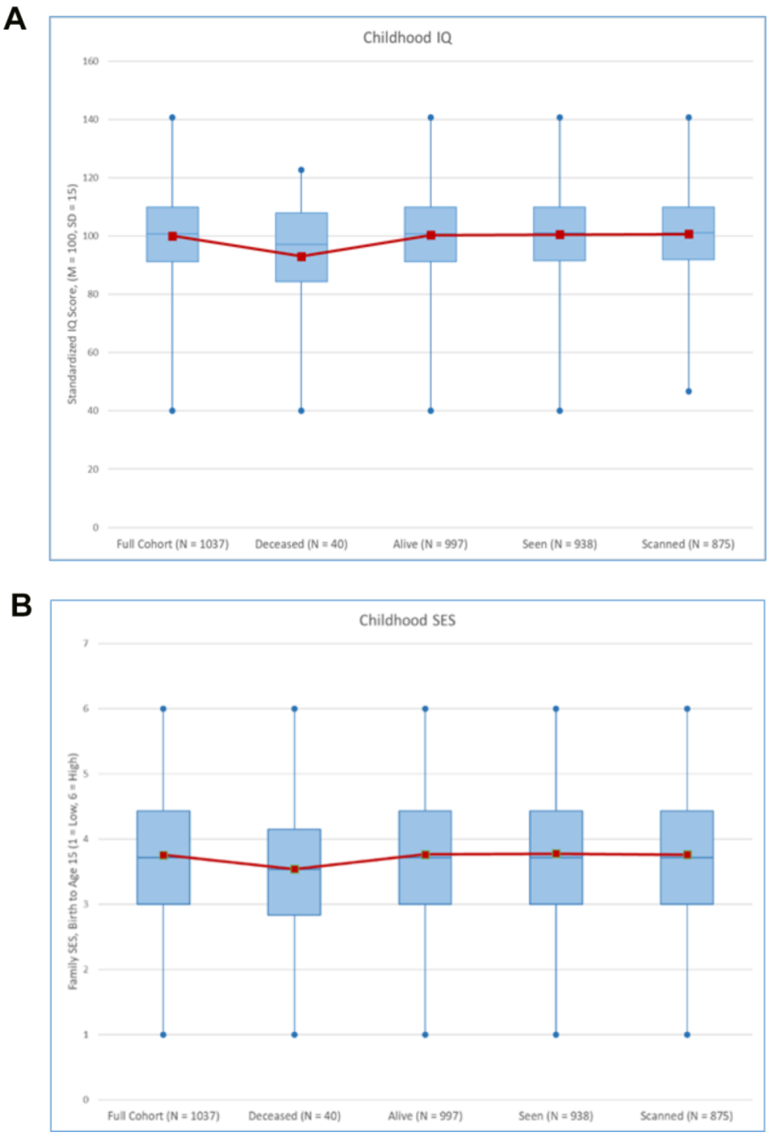

Supplementary Figure 1. We conducted an attrition analysis using childhood neurocognitive functioning (the Wechsler Intelligence Scale for Children–Revised; WISC–R) and socioeconomic status (SES) to determine whether participants in the Phase 45 data collection were representative of the original cohort. (**A**) No significant differences in WISC–R were found between the full cohort, those still alive, or those seen at Phase 45. Those who were deceased by the Phase 45 data collection had significantly lower scores on the WISC–R than those who were still alive (t=2.09, P=.04). (**B**) No significant differences were found between the full cohort, those deceased, those alive, or those seen at Phase 45 on childhood SES.

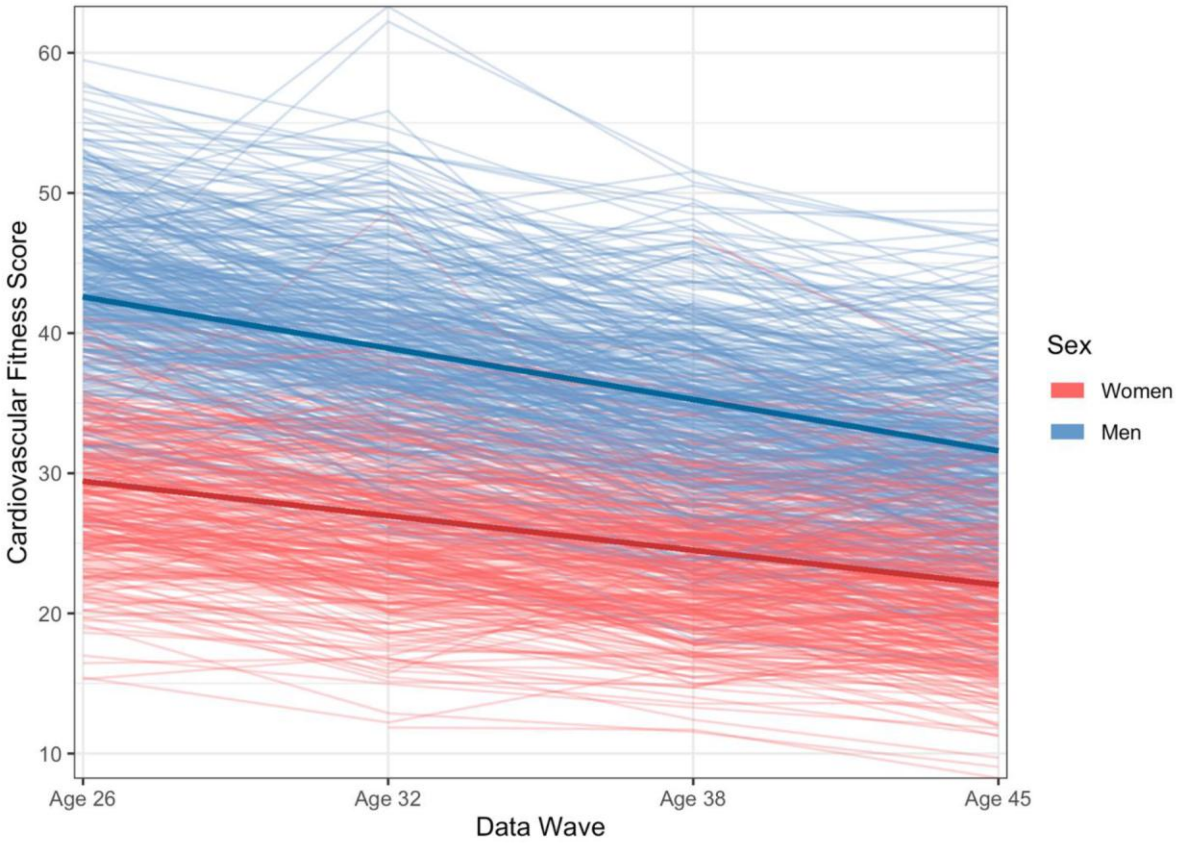

Supplementary Figure 2. Individual VO_2Max scores collected at ages 26, 32, 38, and 45. Individual VO_2Max scores declined over the course of adulthood from age 26 to age 45 in both men and women. Linear growth curve analysis was used to calculate each individual's slope as a measure of the rate of change of VO_2Max over time. Study members had an average VO_2Max score of 26.94 mL/min/kg at age 45, and VO_2Max declined, on average, by 3.16 mL/min/kg between the ages of 26-32, 32-38, and 38-45. VO_2Max = volume of maximum oxygen uptake; mL/min/kg = milliliters per minute per kilogram.

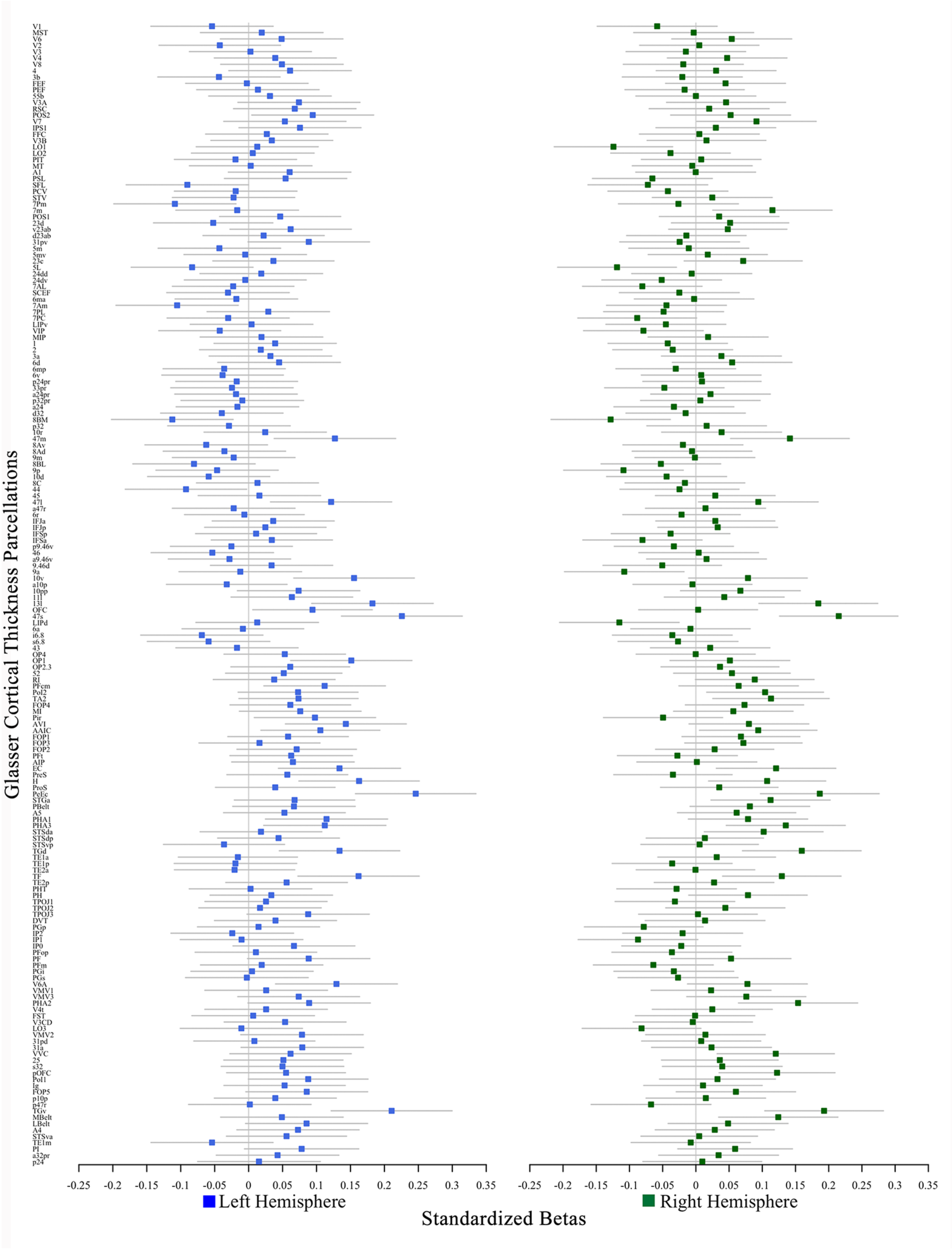

Supplementary Figure 3. Forest plot showing standardized betas for all 360 cortical thickness parcels and cross-sectional fitness in the left and right hemispheres. Confidence intervals displayed are prior to FDR correction. Parcellations defined in Glasser, 2016. FDR = false discovery rate.

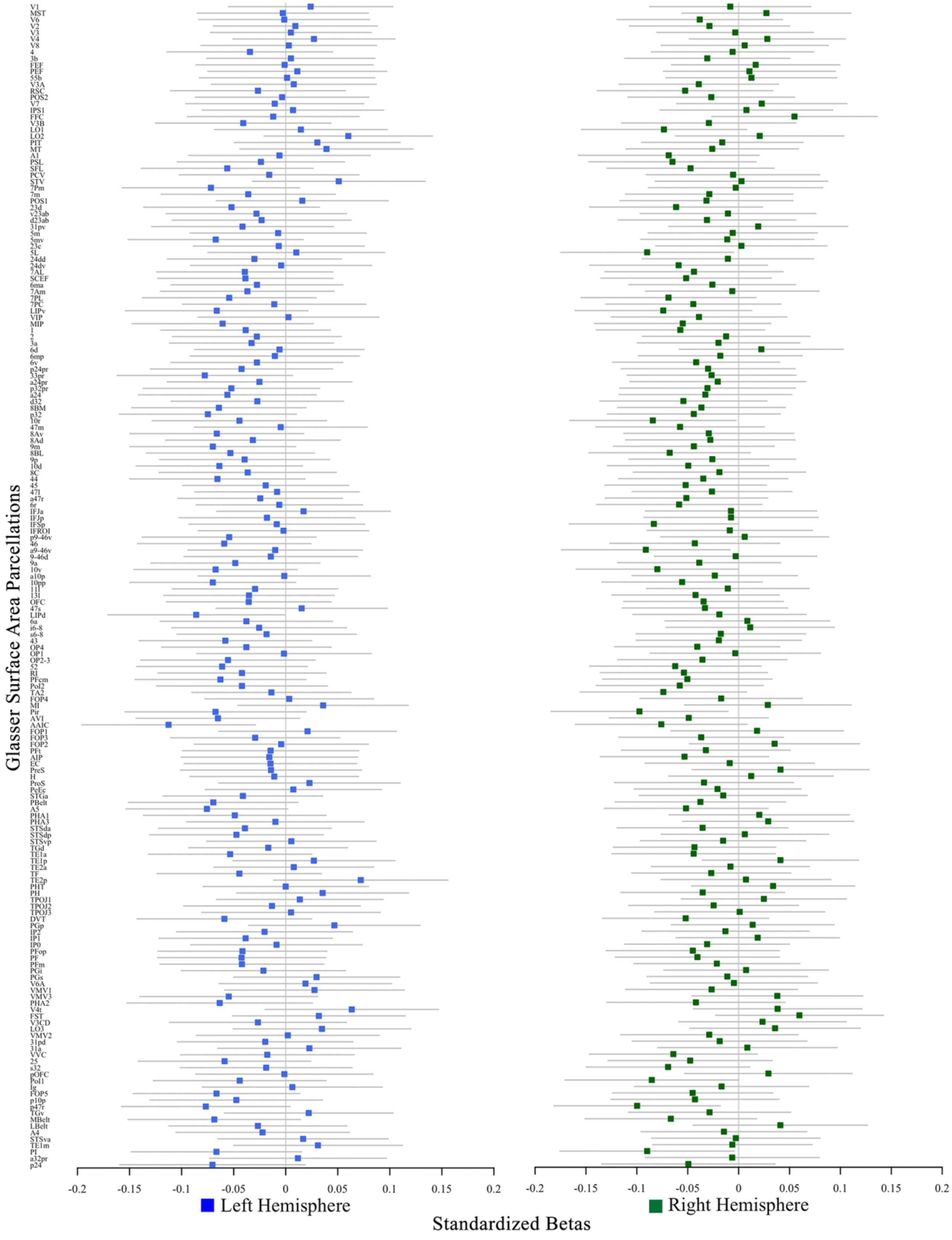

Supplementary Figure 4. Forest plot showing all 360 standardized betas for surface area and cross-sectional fitness analyses in the left and right hemispheres. Confidence intervals displayed are prior to FDR correction. Parcellations defined in Glasser, 2016. FDR = false discovery rate.

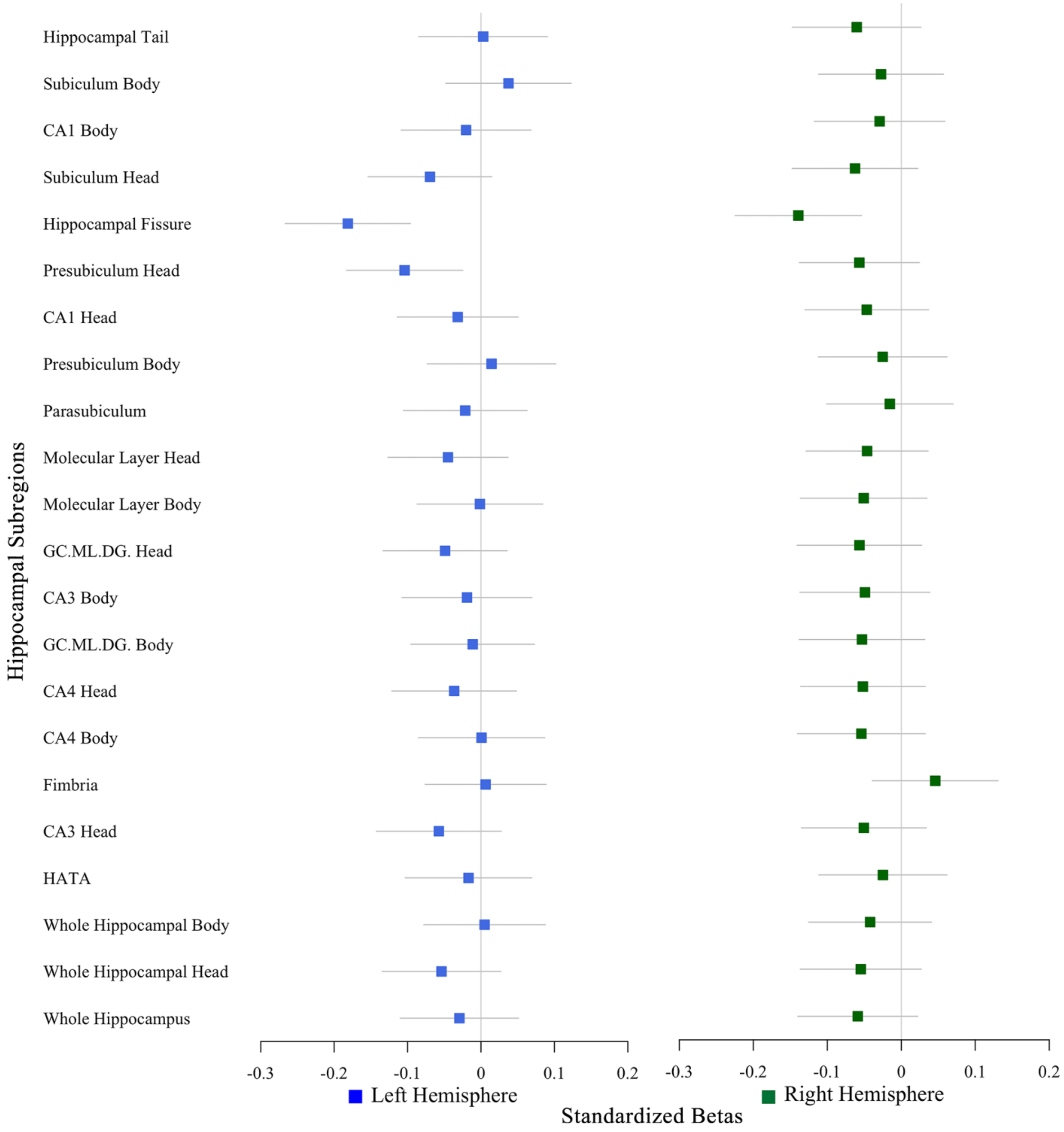

Supplementary Figure 5. Forest plot showing standardized betas for each of the 22 hippocampal subregions and cross-sectional fitness in the left and right hemispheres. Confidence intervals displayed are prior to FDR correction. Parcellations defined in Iglesias, 2015. FDR = false discovery rate.

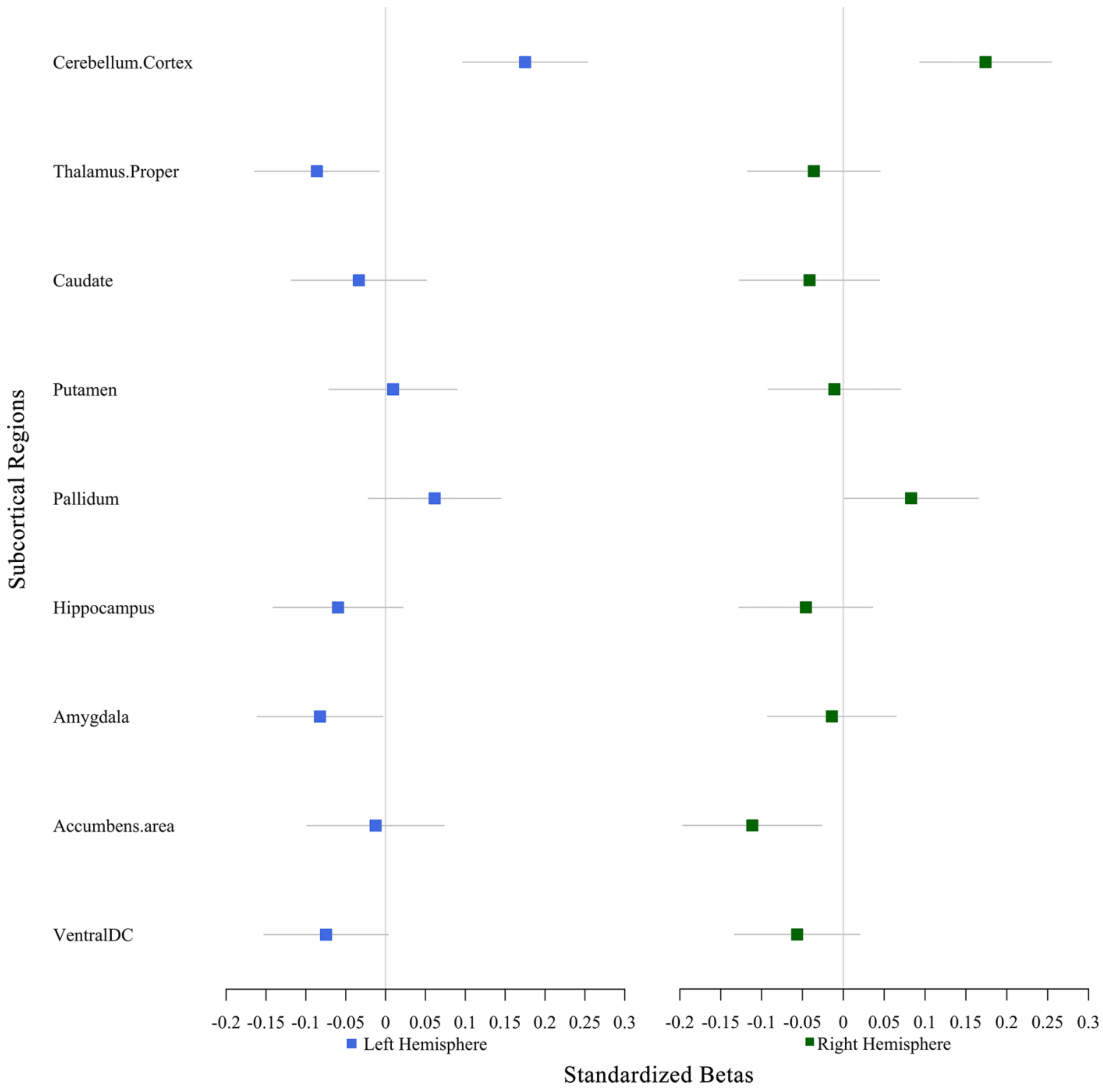

Supplementary Figure 6. Forest plot showing standardized betas for each of the 9 subcortical regions and cross-sectional fitness in the left and right hemispheres. Confidence intervals shown are prior to FDR correction. FDR = false discovery rate.

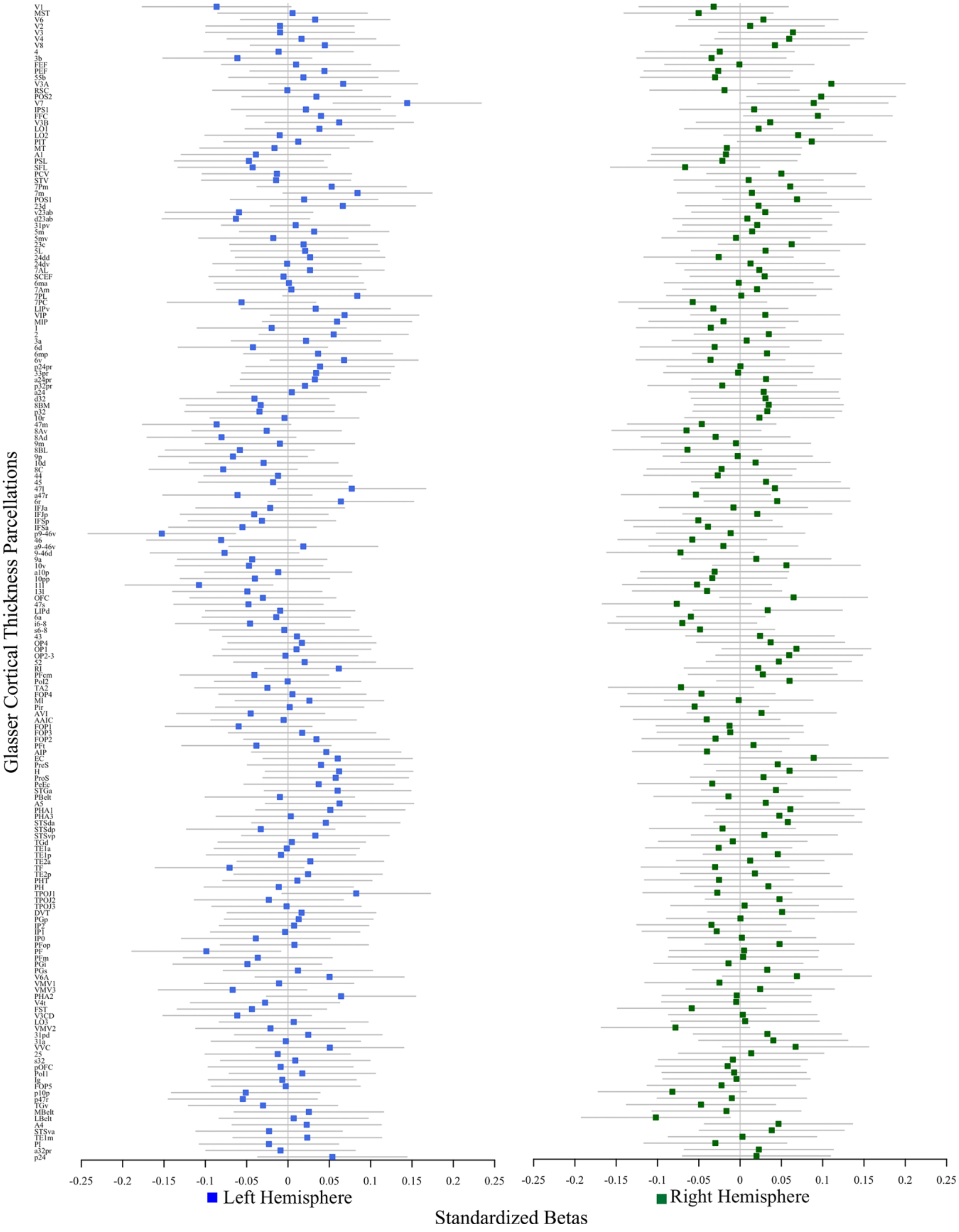

Supplementary Figure 7. Forest plot showing standardized betas for each of the 360 cortical thickness parcels and the rate of change in fitness in the left and right hemispheres. Confidence intervals displayed are prior to FDR correction. Parcellations defined in Glasser, 2016. FDR = false discovery rate.

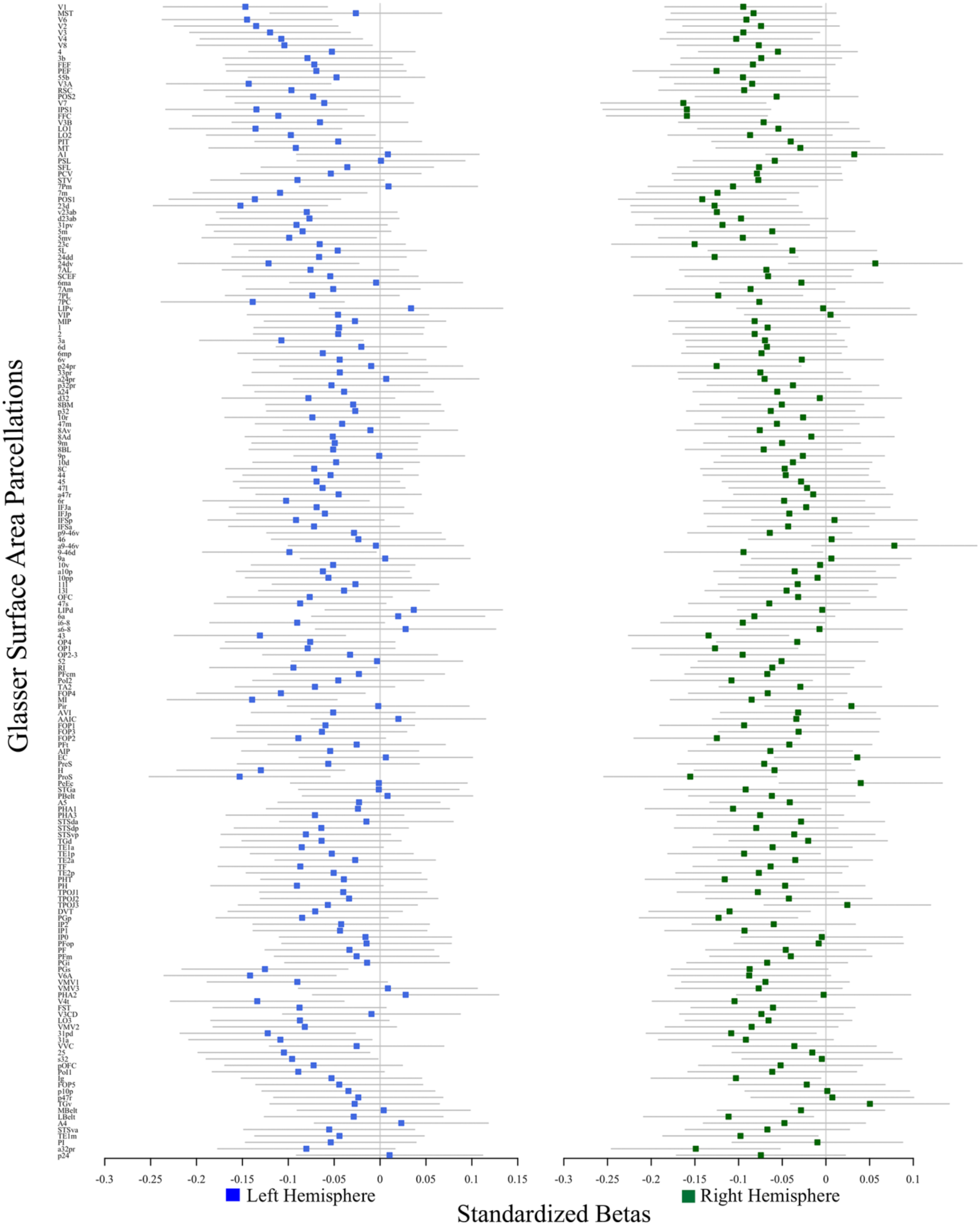

Supplementary Figure 8. Forest plot showing standardized betas for each of the 360 surface area parcels and the rate of change in fitness in the left and right hemispheres. Confidence intervals displayed are prior to FDR correction. Parcellations defined in Glasser, 2016. FDR = false discovery rate.

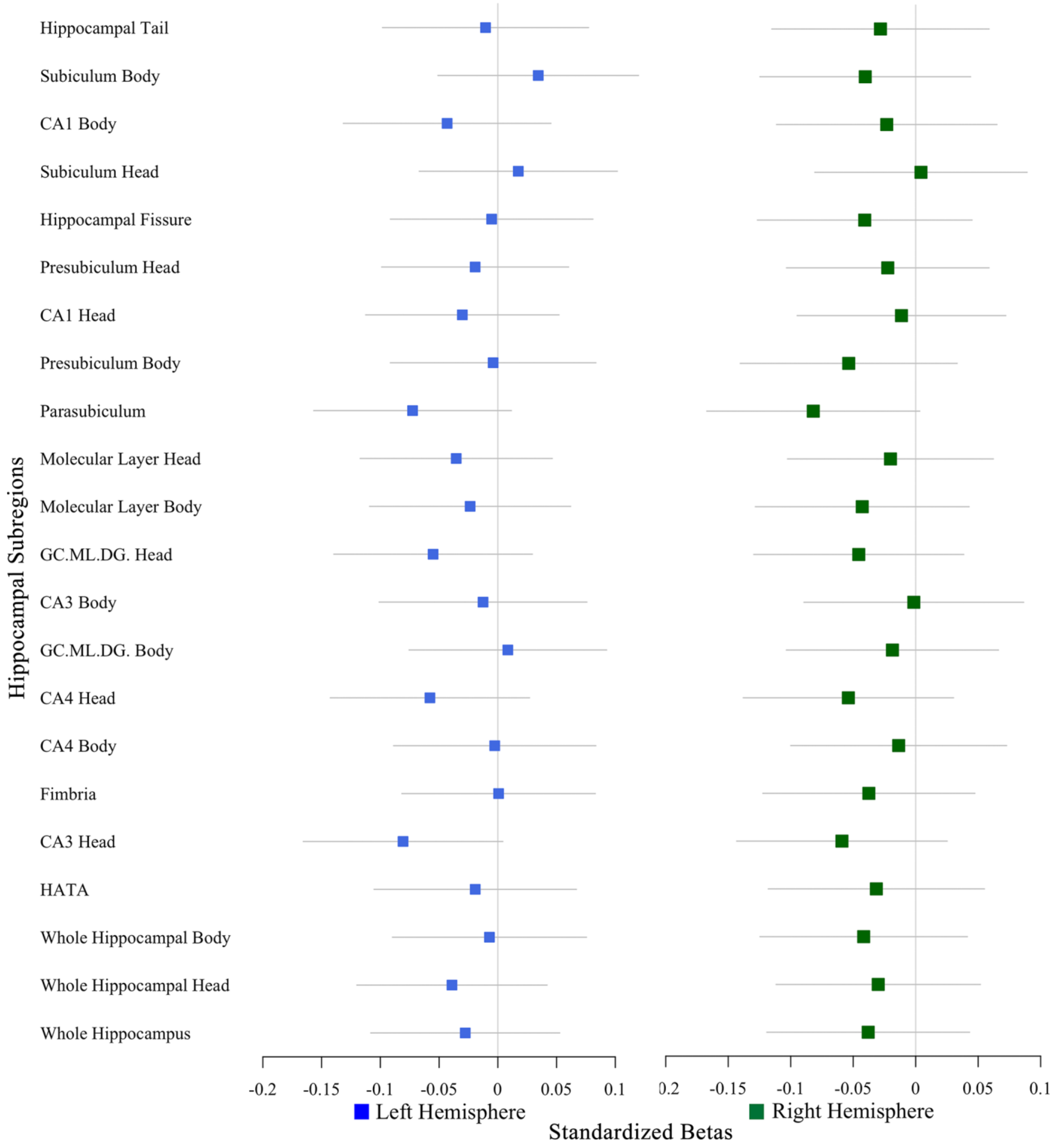

Supplementary Figure 9. Forest plot showing standardized betas for each of the 22 hippocampal subregions and the rate of change in fitness in the left and right hemispheres Confidence intervals shown are prior to FDR correction. Parcellations defined in Iglesias, 2015. FDR = false discovery rate.

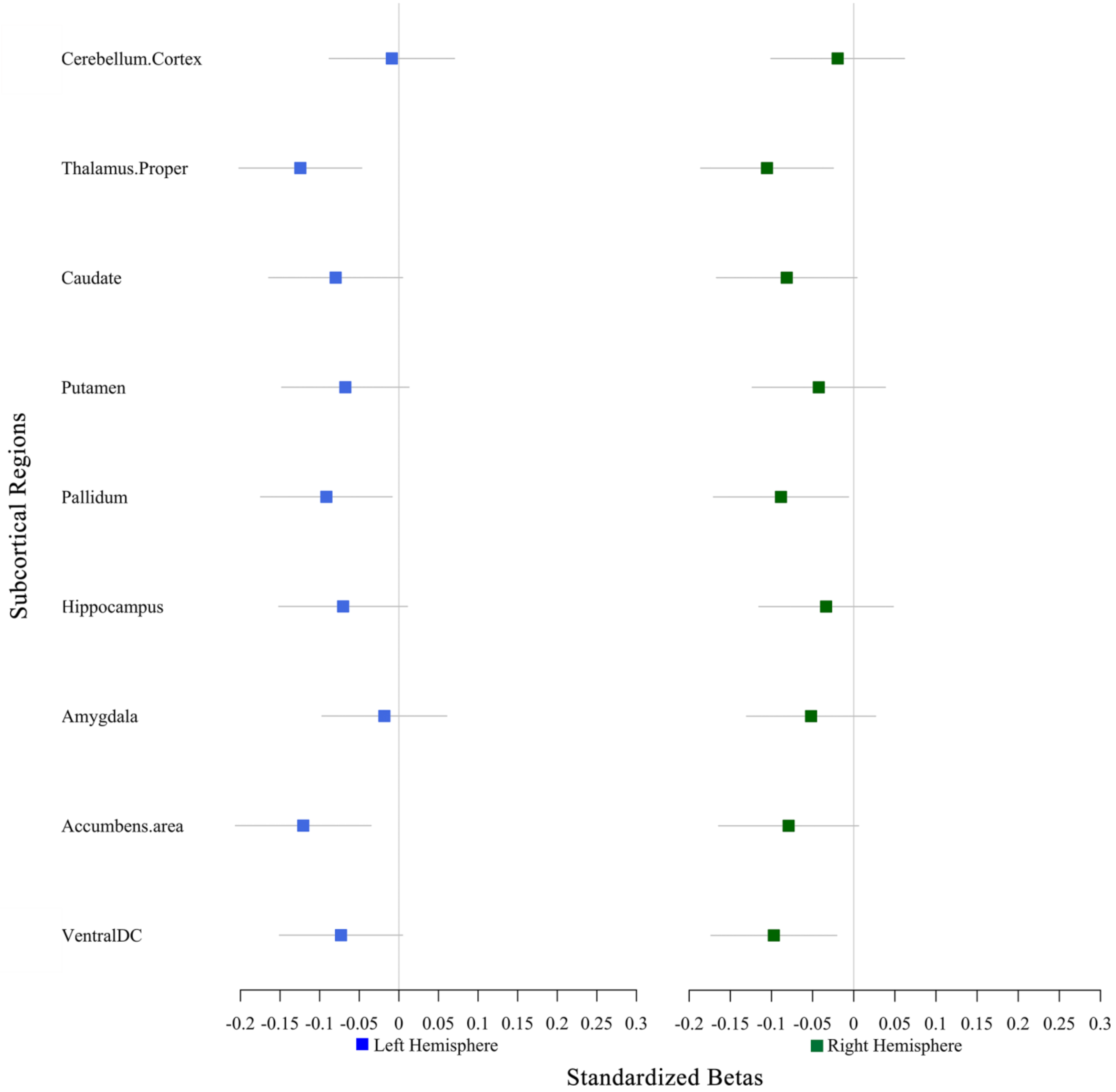

Supplementary Figure 10. Forest plot showing standardized betas for each of the 9 subcortical regions and the rate of change in fitness in the left and right hemispheres Confidence intervals shown are prior to FDR correction. FDR = false discovery rate.

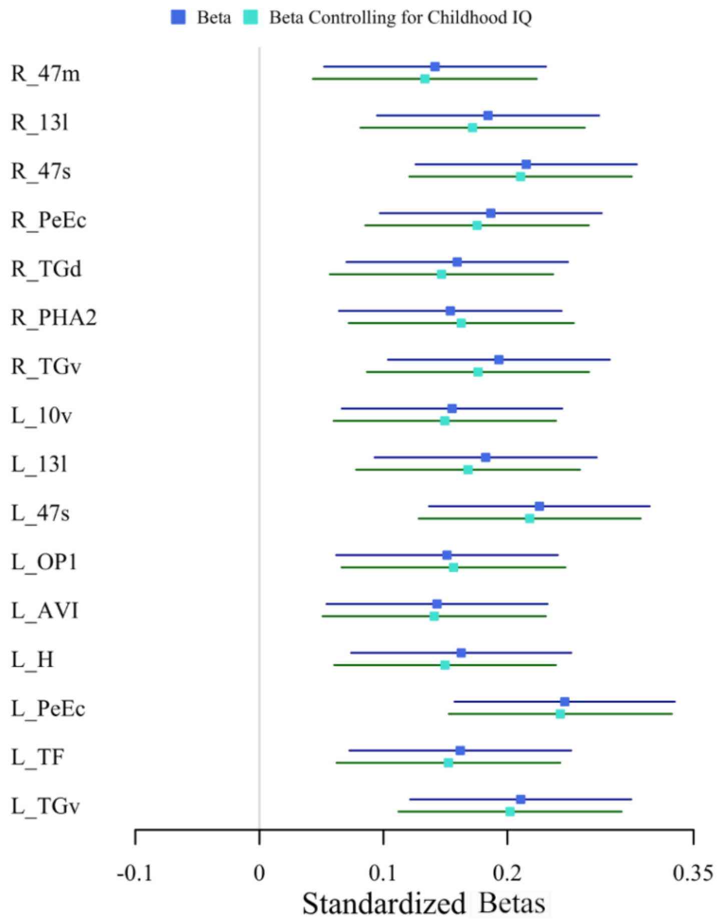

Supplementary Figure 11. Forest plot comparing significant beta coefficients of cortical thickness associated with VO_2Max at age 45 with and without controlling for childhood IQ. The addition of childhood IQ into the analyses did not change the initial results. This suggests that childhood IQ does not explain the association between VO_2max at age 45 and regionally specific cortical thickness in the brain at age 45. Confidence intervals shown are prior to FDR correction. Parcellations defined in Glasser, 2016. VO_2Max = volume of maximum oxygen uptake; IQ = intelligence quotient; L = left hemisphere; R = right hemisphere; FDR = false discovery rate.

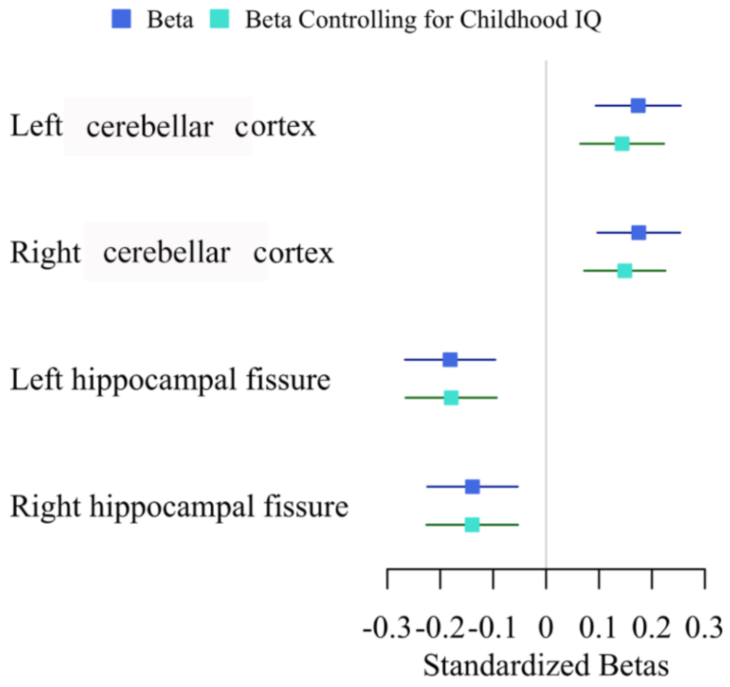

Supplementary Figure 12. Forest plot comparing significant beta coefficients of hippocampal fissure and cerebellar cortex volume associated with the cross sectional VO2Max at age 45 with and without controlling for childhood IQ. The addition of childhood IQ into the analyses did not change the initial results. This suggests that childhood IQ does not explain the association between cardiovascular fitness at 45 and fissure volume or cerebellar volume. Confidence intervals displayed are prior to FDR correction. VO2Max = volume of maximum oxygen uptake; IQ = intelligence quotient; FDR = false discovery rate.
