# Supplementary material for: Is cardiovascular fitness associated with structural brain integrity in midlife? Evidence from a population-representative birth cohort study
Source: Aging (Albany NY). 2020 Oct 21;12(20):20888–914. doi: 10.18632/aging.104112 (PMC7655208; doi:10.18632/aging.104112)
Supplement: Supplementary Tables [file aging-12-104112-s002..pdf]

SUPPLEMENTARY TABLES

Supplementary Table 1. Comparisons of fit statistics for each of the 3 growth curve models fitted to the VO2Max data for ages 26, 32, 38, and 45.

Model	Model Fit	P value	TLI	CFI	RMSEA
Intercept Only	1911.65	<0.001	0.577	0.437	0.54
Intercept and Slope	41.61	<0.001	0.987	0.989	0.095
Quadratic	8.22	0.004	0.987	0.998	0.03

TLI = Tucker-Lewis Index; CFI = Comparative Fit Index; RMSEA = Root mean square error of approximation.

Supplementary Table 2. Sample characteristics of the Dunedin study members at age 45.

Variable	M(SD)	N	t
**VO_2Max (mL/min/kg)**			
Men	31.6(5.91)	414	23.979***
Women	22.04(5.42)	393	
Total	26.94(7.42)	807	
**Rate of Decline in VO_2Max [slope]**			
Men	3.73(.75)	414	24.02***
Women	2.57(.61)	393	
Total	3.16(.9)	807	
**Total Surface Area (cm^2)**			
Men	1949.5(135.5)	414	20.55***
Women	1759.9(126.5)	393	
Total	1850(161.7)	807	
**Average Cortical Thickness (mm)**			
Men	2.57(.09)	414	3.81**
Women	2.55(.09)	393	
Total	2.56(.09)	807	

M(SD) = mean (standard deviation); N = number of participants; t = test statistic; VO_2Max = volume of maximum oxygen uptake; mL/min/kg = milliliters per minute per kilogram; cm^2= centimeters squared; mm = millimeters.
